# Genotypic Characterization of Multidrug-Resistant Pseudomonas aeruginosa Using Whole-Genome Sequencing

**DOI:** 10.1128/MRA.00606-21

**Published:** 2021-08-12

**Authors:** Baha Abdalhamid, Itidal Reslane, Emily Mccutchen, Peter C. Iwen

**Affiliations:** a Department of Pathology and Microbiology, University of Nebraska Medical Center, Omaha, Nebraska, USA; b Nebraska Public Health Laboratory, University of Nebraska Medical Center, Omaha, Nebraska, USA; University of Arizona

## Abstract

Multidrug-resistant Pseudomonas aeruginosa is a serious threat worldwide causing health care-acquired infections and is associated with significant morbidity and mortality. This report describes the draft genome sequences of five multidrug-resistant Pseudomonas aeruginosa strains isolated from human infections.

## ANNOUNCEMENT

Multidrug-resistant Pseudomonas aeruginosa is a common cause of nosocomial infections worldwide and is associated with high morbidity and mortality rates (1, 2). This bacterium can cause surgical site infections, pneumonia, bloodstream infections, and urinary tract infections (1, 2). According to the Centers for Disease Control and Prevention (CDC), more than 32,600 cases of health care-associated infections were caused by multidrug-resistant Pseudomonas aeruginosa in the United States in 2017, which resulted in 2,700 deaths and 767 million dollars of estimated health care costs (2). In this report, we present five draft genome sequences of multidrug-resistant Pseudomonas aeruginosa.

A total of 5 carbapenem-resistant Pseudomonas aeruginosa strains were received from our collaborators in Saudi Arabia. These strains were originally detected from blood, wound, and cerebrospinal fluid samples. All samples were cultured on blood, chocolate, and MacConkey agar plates. All samples were deidentified and therefore did not require institutional review board (IRB) approval. Organism identification and susceptibility testing were performed using a Vitek 2 system (bioMérieux, Marcy I’Etoile, France) as recommended by the manufacturer. All strains were resistant to gentamicin, amikacin, imipenem, meropenem, ceftazidime, cefepime, and ciprofloxacin.

For each isolate, a single pure colony was streaked on blood agar and incubated overnight. A single colony from an overnight blood agar culture was then used for bacterial genomic DNA extraction using the Magna Pure compact instrument and MagNA Pure compact nucleic acid isolation kit (Roche Diagnostics, Indiana, USA). DNA concentration and quality were determined using a Qubit 3.0 fluorometer (Invitrogen, California, USA) and NanoDrop 2000 UV-visible (UV-Vis) spectrophotometer (Fisher, Massachusetts, USA), respectively.

The Illumina MiSeq platform (Illumina, California, USA) was used to perform whole-genome sequencing (WGS) using 200-bp paired-end chemistry. To assess the quality of the WGS, cluster density (600 to 1,200), Phred quality score (QS30, >75%), and >80% of cluster passing filters were used as parameters. A Nextera XT library kit (Illumina) was used to prepare the genomic libraries. All bioinformatics software in this study was used with default parameters unless otherwise specified. FastQC 0.11.9 (https://github.com/s-andrews/FastQC) and Trimmomatic 0.33 (https://github.com/timflutre/trimmomatic) were used to analyze the quality of the sequence reads and trimming, respectively. SPAdes 3.12 (https://github.com/ablab/spades) was used for *de novo* assembly of the quality-trimmed reads. Contigs with fewer than 200 bp were filtered out using BBmap 38.06 (https://github.com/BioInfoTools/BBMap). The quality of the *de novo* assembly was determined using Quast 4.1 (https://github.com/ablab/quast). Prokaryotic Genome Annotation Pipeline (PGAP) 4.9 (https://github.com/ncbi/pgap) was used for annotation. For the 5 strains, the range of the number of contigs was 101 to 201, the range of the total length was 6,444,459 bp to 6,801,376 bp, and the range of the GC content was 65.9% to 66.44% (Table 1).

**TABLE 1:** Characteristics of whole-genome sequencing for five multidrug-resistant Pseudomonas aeruginosa isolates^*a*^

Characteristic	Data for isolate:				
	P2	P8	P13	P28	P30
Genome coverage (×)	36.9	33.0	40.2	37.9	38.9
No. of reads	963,364	871,435	1,061,566	1,000,083	1,026,248
No. of contigs	154	101	151	201	199
Total length (bp)	6,641,132	6,444,459	6,741,877	6,801,376	6,778,388
GC content (%)	66.1	66.4	65.9	66.0	66.0
*N*_50_ (bp)	163,568	187,661	146,757	145,232	159,211
GenBank accession no.	VTFD00000000	VTFA00000000	VTFE00000000	VTFC00000000	VTFB00000000
SRA no.	SRS5311252	SRS5311249	SRS5311253	SRS5311251	SRS5311250
BioSample no.	SAMN12636561	SAMN12636564	SAMN12636560	SAMN12636562	SAMN12636563

a Available at NCBI under BioProject number PRJNA562233.

The data presented in this study are the basis for further studies of antimicrobial resistance and pathogenesis.

### Data availability.

The genomic sequences for these isolates are available at NCBI under the BioProject number PRJNA562233. The GenBank accession numbers, BioSample numbers, Sequence Read Archive (SRA) numbers, number of contigs, *N*_50_ values, and GC contents are provided in Table 1.
